# Amygdalar Corticotropin-Releasing Factor Signaling Is Required for Later-Life Behavioral Dysfunction Following Neonatal Pain

**DOI:** 10.3389/fphys.2021.660792

**Published:** 2021-05-11

**Authors:** Seth M. Davis, Jared T. Zuke, Mariah R. Berchulski, Michael A. Burman

**Affiliations:** ^1^Department of Psychology, University of New England, Biddeford, ME, United States; ^2^Center for Excellence in the Neurosciences, University of New England, Biddeford, ME, United States

**Keywords:** CRF, neonatal pain, fear conditioning, pain sensitivity, CRF antagonists

## Abstract

Neonatal pain such as that experienced by infants in the neonatal intensive care unit is known to produce later-life dysfunction including heightened pain sensitivity and anxiety, although the mechanisms remain unclear. Both chronic pain and stress in adult organisms are known to influence the corticotropin-releasing factor (CRF) system in the Central Nucleus of the Amygdala, making this system a likely candidate for changes following neonatal trauma. To examine this, neonatal rats were subjected to daily pain, non-painful handling or left undisturbed for the first week of life. Beginning on postnatal day, 24 male and female rats were subjected to a 4-day fear conditioning and sensory testing protocol. Some subjects received intra-amygdalar administration of either Vehicle, the CRF receptor 1 (CRF_1_) receptor antagonist Antalarmin, or the CRF receptor 2 (CRF_2_) receptor antagonist Astressin 2B prior to fear conditioning and somatosensory testing, while others had tissue collected following fear conditioning and CRF expression in the CeA and BLA was assessed using fluorescent *in situ* hybridization. CRF_1_ antagonism attenuated fear-induced hypersensitivity in neonatal pain and handled rats, while CRF_2_ antagonism produced a general antinociception. In addition, neonatal pain and handling produced a lateralized sex-dependent decrease in CRF expression, with males showing a diminished number of CRF-expressing cells in the right CeA and females showing a similar reduction in the number of CRF-expressing cells in the left BLA compared to undisturbed controls. These data show that the amygdalar CRF system is a likely target for alleviating dysfunction produced by early life trauma and that this system continues to play a major role in the lasting effects of such trauma into the juvenile stage of development.

## Introduction

Painful neonatal procedures are commonplace among infants admitted into neonatal intensive care units (NICUs), and many procedures (e.g., heel lance, intravenous and/or arterial line insertions, endotracheal suctioning, etc.) are performed without the use of analgesics despite being rated as moderately to very painful (Porter et al., 1997; Roofthooft et al., 2014; Fitzgerald, 2015). However, the long-term negative effects of early life pain in humans are now well-established. Children who spend more time in the NICU are more likely to develop depression, anxiety, and altered pain sensitivity/susceptibility to chronic pain states (Taddio et al., 1997; Porter et al., 1999; Anand and Scalzo, 2000; Hummel and Puchalski, 2001; Simons et al., 2003; Grunau et al., 2006; Brummelte et al., 2012; Mooney-Leber and Brummelte, 2017; Walker, 2019; Williams and Lascelles, 2020). However, from human epidemiological data alone, it is not clear whether later-life dysfunction following NICU admission is the result of the painful procedures that occur in the NICU, concomitant factors such as lack of caregiver contact, or underlying health concerns and/or prematurity that led to placement in the NICU. Moreover, the mechanisms by which early life pain and stress lead to later consequences remain unclear, which is an obstacle for both identifying and treating at-risk individuals.

Animal models can be used to experimentally manipulate non-human animals to better understand the impact of neonatal pain on later-life behavior and avoid or control these confounding factors. One common finding is that neonatal pain influences later-life pain sensitivity (Butkevich et al., 2016; Davis et al., 2018; Mooney-Leber et al., 2018; de Carvalho et al., 2019; Mooney-Leber and Brummelte, 2019; Davis and Burman, 2020), with the general consensus that neonatal pain increases pain sensitivity in rodents, particularly following a second “activating” stressor. In addition, neonatal pain has sometimes resulted in the decrease of subsequent fear and anxiety in rodents (Davis et al., 2018, 2020; Zuke et al., 2019; Xia et al., 2020), although the literature is somewhat inconsistent (Anand et al., 1999).

Although the mechanisms of these effects remain unclear, we have previously shown that changes in corticotropin-releasing factor (CRF) activity in the amygdala may play a role (Zuke et al., 2019), at least in male rodents, similar to effects reported in the visceral hypersensitivity literature (Prusator and Greenwood-Van Meerveld, 2016a). In adult rodents, CRF-expressing cells in the amygdala are involved with behavioral and sympathetic nervous system responses to pain and threatening environments (Beggs et al., 2012; Rouwette et al., 2012) and are altered during the chronification of pain in arthritis and neuropathic pain models (Ulrich-Lai et al., 2006; Ji et al., 2007; Neugebauer et al., 2020). In neonatal rodents, elevated amygdalar CRF has been observed following neonatal pain or cold stress (Brunson et al., 2001; Zuke et al., 2019). Neonatal maternal separation (Pihoker, 1993; O’Malley et al., 2011) or pre-weaning odor-shock pairings (Prusator and Greenwood-Van Meerveld, 2016b) have also been shown to increase central nervous system CRF expression later in adult rats. Moreover, there is growing evidence that there may be significant sex differences in the neuro/endocrine response to neonatal trauma (Farrell et al., 2016; Prusator and Greenwood-Van Meerveld, 2016a, b; Goodwill et al., 2019; Bath, 2020; Louwies and Greenwood-Van Meerveld, 2020).

Amygdalar CRF have also been implicated with regard to fear and anxiety in rodents (Deak et al., 1999; Takahashi, 2001; Zorrilla et al., 2002). Specifically, it has been shown that intra-amygdalar CRF impaired fear extinction (Abiri et al., 2014) and amygdala CRF levels positively correlate with contextual freezing duration (defined as low responders vs. high responders) (Lehner et al., 2008). In developing subjects, Liu et al. (2011) demonstrated that juvenile rats (Postnatal Day; PND 10) subjected to a painful gastric irritation procedure and tested at 8–10 weeks of age displayed higher anxiety- and depressive-like behaviors as well as elevated hypothalamic and amygdaloid CRF expression. The anxiety- and depressive-like behaviors were reversed by treatment with the CRF receptor 1 (CRF_1_) antagonist, Antalarmin.

Corticotropin-releasing factor has two main targets. CRF binds primarily to CRF_1_. Although CRF receptor 2 (CRF_2_) primarily binds urocortins, it also has modest affinity for CRF. These two G_*s*_-coupled receptors appear to play opposing roles in behavior (Chang et al., 1993; Zorrilla et al., 2002; Baiamonte et al., 2014). The CRF_1_ antagonist Antalarmin has been shown to attenuate inflammatory pain (Webster et al., 2002) and reduce stress-induced visceral hypersensitivity (Greenwood-Van Meerveld et al., 2005; Myers et al., 2005; Larauche et al., 2012), while the selective CRF_1_ antagonist NBI27914 reversed tactile hypersensitivity in arthritic rats (Ji et al., 2007). Antalarmin has also been shown to reduce fear conditioning responses in rats defined as “high anxiety” (Skórzewska et al., 2019), while both oral administration and intra-amygdalar infusions of the selective CRF_1_ antagonist DMP696 reduced contextual freezing with no effect on fear acquisition (Hubbard et al., 2007). Moreover, the CRF_1_ antagonist CP-154526, but not the CRF_2_ antagonist aSVG30, reduced visceral pain-related behaviors in rats (Nijsen et al., 2005). Although it is less known, CRF_2_ may play an opposing role in fear and anxiety in that CRF_2_ blockade results in enhanced anxiogenic and conditioned fear responses (Radulovic et al., 1999; Skórzewska et al., 2011).

We seek to examine whether lasting changes in CeA CRF expression are a potential mechanism of the lasting effects of neonatal pain on later behavior. To accomplish this, we will determine whether the CeA CRF system remains altered in juvenile rats following neonatal pain by assessing both CRF mRNA expression and the activation of immediate early gene c-fos, which is sometimes implicated as a marker of neuronal activity in the amygdala immediately following stressful and painful stimuli (Nakagawa et al., 2003; Baker and Kim, 2004; Allen et al., 2021). In addition, we will observe the effects of intra-CeA CRF_1_ and CRF_2_ antagonists on fear conditioning and the subsequent tactile hypersensitivity in three separate experiments. Experiment 1 assesses the effects of adolescent intra-CeA Antalarmin (A CRF_1_ antagonist) in rats that received neonatal pain. Experiment 2 assesses the effects of adolescent intra-CeA Astressin 2B (A CRF_2_ antagonist) in rats that received neonatal pain. Experiment 3 examines the adolescent expression of CRF and c-fos mRNA using fluorescent *in situ* hybridization in rats that received neonatal pain. This study is among the first to demonstrate a sex-dependent and lateralized role of CeA CRF in somatic hypersensitivity that results from an early life stressor closely resembling the NICU experience.

## Materials and Methods

### Subjects

Male and female Sprague Dawley rats were bred in-house using a protocol previously described in Davis et al. (2018) and Zuke et al. (2019). Experiment 1 used a total of 127 subjects from 48 litters (26 neonatally manipulated and 22 undisturbed), Experiment 2 used a total of 111 subjects from 35 litters (20 neonatally manipulated and 15 undisturbed), and Experiment 3 used 14 litters (7 neonatally manipulated and 7 undisturbed). Experimental *N*’s were determined via an *a priori* power analysis which indicated a suggested *N* of nine rats per group in order to achieve a high effect size. All litters were housed in 43 cm × 44 cm × 20 cm closed-environment cages (Innovive, San Diego, CA, United States). On PND 1, pups were removed from their mother, placed on a heating pad, sexed, marked via crystal violet stain, and culled to no more than 10 rats per litter (five males and five females when possible). In an effort to keep undisturbed litters the least disturbed as possible, body weights during the neonatal period were not collected. Pups were weaned on PND 21 and lived with their same-sex littermates (approximately five per cage). No more than one same sex littermate was assigned to each experimental group (with the experimental group defined as a combination of neonatal treatment, sex, and age at testing). In the rare situation where that was violated, the data from the two subjects were averaged. All rats were maintained on a 12:12 light/dark cycle with lights on at 07:00. Food and water were available *ad libitum*, and at the end of experimentation, rats were euthanized via pentobarbital overdose and brains were collected to check for placements. All rats were treated in accordance to the NIH *Guide for the Care and Use of Laboratory Animals* (2011) and approved by the University of New England’s Institutional Animal Care and Use Committee (IACUC).

### Neonatal Pain

**FIGURE 1:**
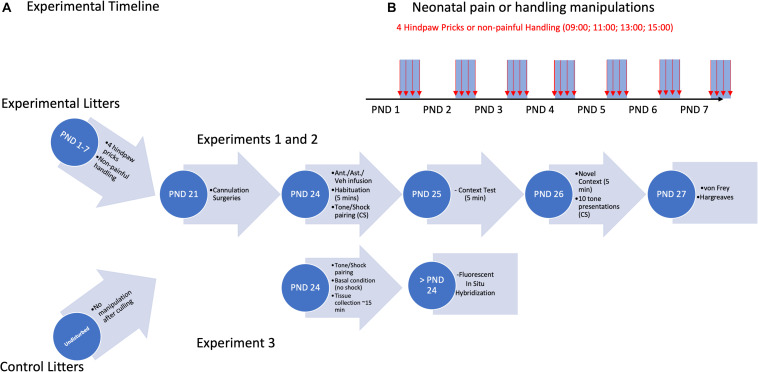
Experimental timeline **(A)** for cannulation Experiments 1 and 2, and CeA/BLA CRF expression Experiment 3. Overview of neonatal pain/handling manipulations **(B)** for all three Experiments.

The procedure was similar to that previously described (Davis et al., 2018). Briefly, on PNDs 1–7, rats were placed on a heating pad and received either a left hindpaw prick (using a 24-G needle tip every 2 h, four times per day starting at 09:00) or non-painful handling by touching the left hindpaw with the index finger. See Figure 1 for an experimental timeline.

### Surgery

For Experiments 1 and 2, on PND 22 rats underwent bilateral cannulation targeting the central nucleus of the amygdala. Rats were anesthetized using isoflurane gas (Patterson, Greeley, CO, United States) and mounted on a stereotaxic apparatus (Stoelting, Wood Dale, IL, United States). The isoflurane was mixed with oxygen in a calibrated vaporizer (VetEquip, Livermore, CA, United States) at a range of 2.5–3% with a flow rate of approximately 1.5 bar. Flow rate and mixture percentage were adjusted on an individual basis to ensure there were no reflexive responses as well as periodic breathing rate assessments. Guide cannulae (Plastics One, Roanoke, VA, United States) were placed in males: –2.00 mm A/P, ± 3.6 mm M/L, –7.00 mm and females: –2.00 mm A/P, ± 3.8 mm M/L, –7.00 mm from bregma and secured using skull screws and acrylic dental cement. All rats received 0.03 mg/kg buprenorphine and allowed to fully recover. Dummy cannulae (Plastics One, Roanoke, VA, United States) were inserted, and daily health checks were performed until testing.

### Infusions

Immediately prior to fear conditioning (see below), rats were infused, while they were fully conscious and freely moving, with a CRF_1_ or CRF_2_ antagonist [experiment 1: Antalarmin (2.5 mg/ml), experiment 2: Astressin 2B (1 mg/ml)] or Vehicle (saline) using a microinfusion pump (Zorrilla et al., 2002; Henry et al., 2006; Gondré-Lewis et al., 2016). Rats received a 0.5-μl volume over 2 min at a rate of 0.25 μl/min. Once infusions were complete, the injector cannulae were left in place for 1 min to allow for diffusion.

### Apparatus

Fear conditioning was performed in four Startfear chambers (Harvard Apparatus/Panlab model #58722) with two separate contextual cues that differed in shape (square vs. circle), color (black vs. white walls), and scent (70% ethanol vs. 1% ammonia). Tactile allodynia was measured by using the up/down technique (Chaplan et al., 1994) with von Frey monofilaments (North Coast Medical, Gilroy, CA, United States) of varying gauges (equating to 0.4–15 g pressure). Thermal hyperalgesia was assessed using a Hargreaves apparatus (Ugo Basile Plantar Test model #7371, Collegeville, PA, United States).

### Fear Conditioning

On PNDs 24, all rats underwent a mild fear conditioning protocol, similar to that previously described (Deal et al., 2016; Davis et al., 2018). On Day 1 of testing, rats received an infusion (Experiments 1 and 2) and were placed in their preassigned (counterbalanced) fear conditioning chamber (FCC) and the program was initiated. The percent of time spent freezing during the first 5 min was recorded (Habituation). Following the habituation period, a 67-dB tone conditioned stimulus (CS) was presented for 10 s, immediately followed by a 2-s 0.3-mA foot shock serving as the unconditioned stimulus (UCS). There were 10 tone-shock pairings. Experiment 3 utilized this procedure on PND 24 for a subset of subjects. However, these subjects received no infusions and were euthanized 15 min after this procedure.

The following day (Experiments 1 and 2), rats were placed in the same FCC and the percent freezing for 5 min was recorded (Context). The third day, rats were placed in a different FCC with different contextual cues than their prior FCC and freezing for the first 5 min was recorded (Novel Context). Subsequently, there were 10 presentations of the original CS (67-dB tone) every 30 s, and the percent freezing for the 10-s period during each CS presentation was recorded and averaged (AVG Tone).

### Tissue Collection

All subjects designated for Experiment 3 were euthanized with a volume of 0.25 ml pentobarbital (390 mg/ml) and 4% paraformaldehyde (PFA) cardiac perfusion on PND 24. Brains were collected and subsequently postfixed for 24 h in 4% PFA. Additionally, for Experiment 3, due to the immediate enhancement of c-fos expression following pain and stress, we chose to collect fear-conditioned subjects’ brains 15 min post-fear conditioning; this gave experimenters enough time to relocate fear-conditioned animals from behavioral testing equipment to surgical space. All animal euthanasia was consistent with the American Veterinary Medical Association procedures. After the 24-h postfix, brains were cryoprotected in 30% sucrose. When cryoprotection was finished, brains were embedded in Tissue-Tek O.C.T. Compound (Sakura Finetek) and flash frozen using liquid nitrogen. Brains were then stored at –80°C prior to cryosectioning at 15 μm onto Superfrost Plus microscope slides. Emphasis was placed to identify two sections between –1.8 and –2.4 bregma for consistency between subjects and optimal amygdala presentation. These sections were stored at –80°C until application of our RNAscope^®^ Fluorescence *in situ* hybridization (FISH) protocol (approximately 1 month).

### Somatosensory Testing

Twenty-four hours after the Novel Context and Tone tests, rats in Experiments 1 and 2 underwent somatosensory testing identical to that reported in Davis et al. (2018, 2020). Tactile allodynia of the left hindpaw was measured using the up–down method with von Frey monofilaments ranging from 2 to 15 g as previously described by Chaplan et al. (1994). Upon completion, rats were subjected to the Hargreaves measure of thermal hyperalgesia, which involved placing the rat’s left hindpaw over a diode that increased infrared light intensity until the rat removed its hindpaw from the thermal stimulus. These methods were previously described in Davis et al. (2018).

### Placement Verification

**FIGURE 2:**
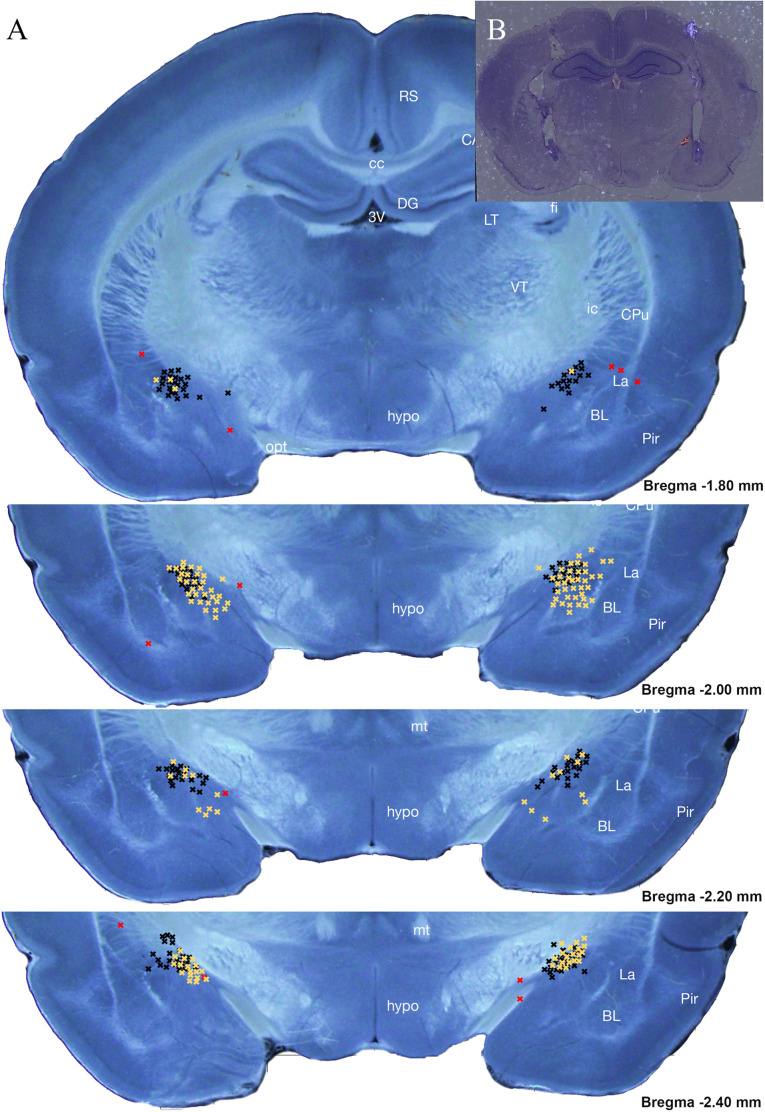
Intracranial cannula placement diagram **(A)** and an example section **(B)** of histological analysis for subjects within Experiments 1 (black) and 2 (yellow) placement location of Antalarmin, Astressin 2B, or Vehicle infusion into the CeA. A total of 228 subjects were analyzed with only 18 from Experiments 1 and 5 from Experiment 2 determined to be misses. Each guide cannula “hit” viewed ended at one of the black X’s seen on the stitched atlas image between –1.80 and –2.40 mm away from Bregma. Any X’s categorized as “misses” are seen in red and ended at a point outside of this region of interest depicted (Bregma –1.80 to –2.40 mm). Misses were excluded from data analysis. A miss was categorized as >1 mm away from the CeA. Neonatal brain images courtesy of Khazipov et al. (2015).

Once behavioral testing was completed, all rats were perfused with 4% PFA and brains were collected. Following a 24-h postfixation period and a 2–3-day saturation in 30% sucrose as a cryoprotectant, brains were frozen using liquid nitrogen and were then sectioned using a freezing stage cryostat with the target sections being the beginning to end of cannula tracts. All sections were then stained via cresyl violet and visualized using light microscopy to ensure that the target (CeA) was within 1 mm of the end of the cannula guide tract (see Figure 2). This was done using two independent observers. Rats that had a missed target on one or both sides were removed from further analysis. This process removed 13.2% of subjects from Experiment 1, totaling 17 subjects (Antalarmin—three female Pain, one female Handled, two male Handled, two male Undisturbed; Vehicle—two female Pain, two male Pain, two female Handled, one female Undisturbed, two male Undisturbed) and 4% of subjects from Experiment 2, totaling five subjects (three Undisturbed vehicle females, one Undisturbed Astressin 2B male, one Undisturbed vehicle male).

### Fluorescence *in situ* Hybridization

All FISH was performed using a commercially available system [RNAscope; Advanced Cell Diagnostics (ACD)] and utilizing probes targeting CRF (product number: 318931) and c-fos (product number: 403591-C2). Our protocol was developed using the RNAscope Multiplex Fluorescent Reagent Kit v2 user manual (document number: 323100-USM), manufacturer technical note regarding tissue detachment, manufacturer modifications for fixed frozen tissue (ACD), and our previous work (Zuke et al., 2019). In the concluding steps of our protocol, DAPI was applied to brain sections as a counterstrain for region identification.

RNAscope assays were conducted in batches. Each batch contained one section from each condition (sex × neonatal treatment × juvenile treatment) collected from the same litter on the same day (PND 24). Additionally, RNAscope batches were duplicated such that each brain was stained twice, with neighboring sections from the same brain being run in separate batches. Lastly, neighboring sections from the same brain were averaged across quantification measures to create an average for each subject (called subject average), which was used for all subsequent analyses. Per manufacturer instructions (RNAscope; ACD) to ensure consistency between subsequent FISH batches, each batch consisted of additional sections processed with either positive control probe [product number: 320891; containing Polr2a (channel 1), PPIB (channel 2)] or negative control probe [product number: 320871; containing DapB gene accession EF191515 from the SMY strain of Bacillus subtilis (channels 1 and 2)].

**FIGURE 3:**
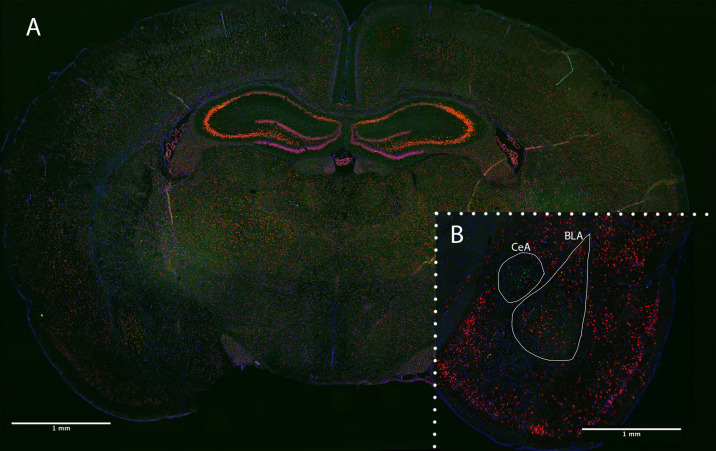
Coronal brain section of PND 24 rat following RNAscope FISH. **(A)** Optimized positive control section staining Polr2a (green), PPIB (red), and DAPI (blue). **(B)** Experimentally stained section staining CRF (green), c-fos (red), and DAPI (blue). Quantified amygdala regions (BLA and CeA) indicated in a female, fear-conditioned, handled experimental section.

**FIGURE 4:**
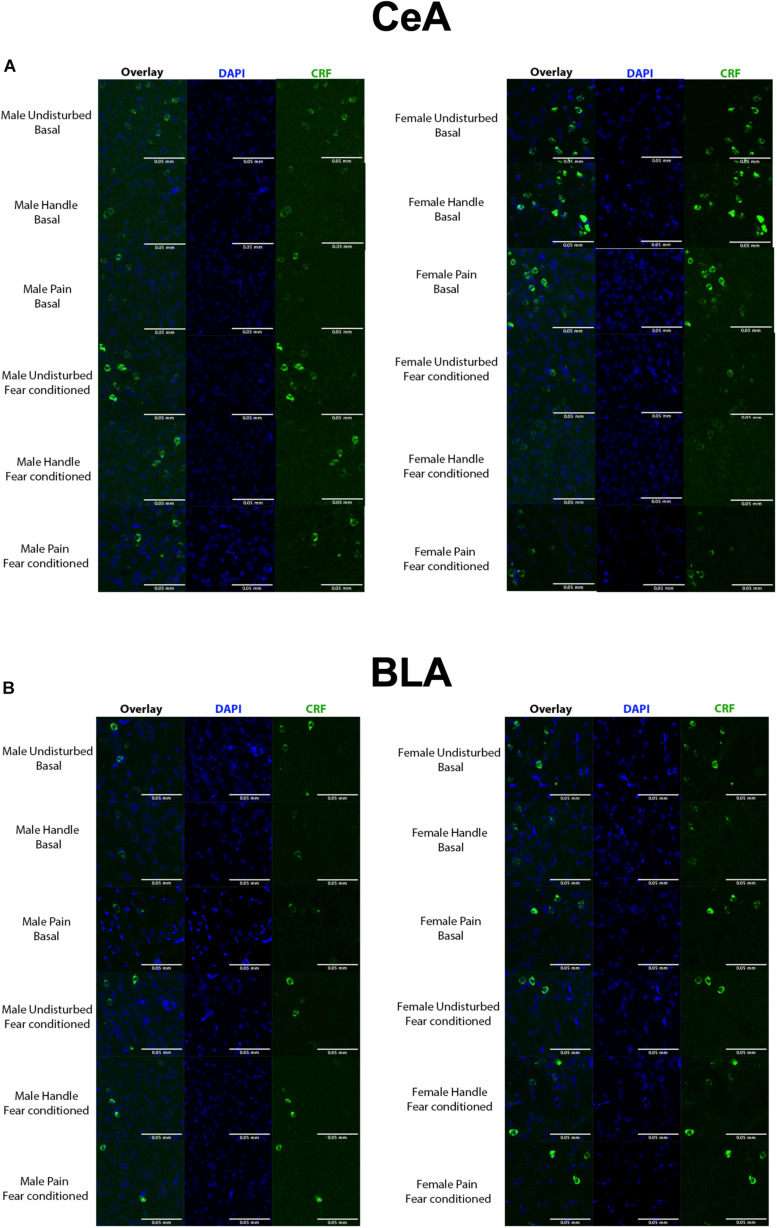
**(A)** Quantified FISH image samples from each represented group within the CeA. Green = CRF, blue = DAPI. **(B)** Quantified FISH image samples from each represented group within the BLA. Green = CRF, blue = DAPI. N’s ranged 4–7 subjects per group.

Fluorescence multiplex imaging was done between 2 days and 2 weeks after FISH. All quantified images and channels were taken under the same magnification (20×) and exposure settings (CRF: 1/8.5s, c-fos: 1/15) on a Keyence BZ-X710 microscope. The DAPI channel exposures varied somewhat between sections due to the inherent variability in DAPI application. Sections were overlaid and stitched for analysis using image merge software provided with Keyence BZ-X710 microscope (Keyence). Images were analyzed using the FIJI package of NIH’s open-source image analysis software ImageJ (Schindelin et al., 2012; Schneider et al., 2012). Images were first subjected to channel separation, and the DAPI channel was used to Identify regions of quantification (BLA and CeA). The DAPI channel was then dilated and used to create a cell mask for our amygdala regions. The number of cells containing CRF, c-fos, or both (colocalized) in the BLA and CeA were collected (see Figure 3). Additionally, luminance of CRF straining in CRF-containing cells was accessed in these regions via the averaging of grayscale values. All images were subject to the same thresholding, processing, and quantification methods (see Figure 4).

### Experimental Design and Analysis

Data were analyzed with IBM SPSS version 25 using mixed-model MANOVAs or ANOVAs with Greenhouse–Geisser corrections where sphericity was violated. No more than one same-sex littermate was assigned to each experimental group. In the rare situation where that was violated, the data from the two subjects were averaged. Data are reported as mean ± SEM, and a *p*-value of ≤0.05 was considered statistically significant, while *p*-values between 0.05 and 0.10 were considered trends toward significance.

For Experiments 1 and 2, independent variables included drug (Vehicle or Antalarmin/Astressin 2B), sex (male or female), and neonatal condition (Undisturbed, Handled, or Pain). Dependent variables included three contextual freezing variables (Habituation, Context, and Novel Context) which were run as repeated-measure ANOVAs; tone freezing variables (Avg. Tone) which were analyzed as univariate ANOVAs; and the somatosensation variables (mechanical and thermal withdrawal thresholds) which were also analyzed as univariate ANOVAs. A Tukey’s *post hoc* analysis was performed when there were more than two levels of a variable to compare. No more than one same-sex littermate was assigned to each experimental group. Outlier analyses were performed prior to data analyses, and data points were removed if a subject’s data were more than 2.5 standard deviations from the mean.

For Experiment 3, independent variables included sex (male or female), neonatal condition (Undisturbed, Handled, or Pain), and juvenile condition (basal, fear conditioned). Dependent variables include number of CRF expressing cells and average luminance of CRF-expressing cells across hemispheres and in both regions (CeA and BLA). First, repeated-measure ANOVAs were conducted for all CRF measures and regions to determine main effects or interactions of hemisphere. If an effect or interaction was found, subsequent univariate ANOVAs were conducted on all CRF measures for each side independently. Furthermore, if an effect of sex or juvenile condition was observed, then additional univariate ANOVAs were conducted separating those respective variables. Lastly, and where applicable, *post hoc* (Dunnett) tests were performed to assess the differences between the treated and undisturbed neonatal conditions.

## Results

### Experiment 1: Effects of Neonatal Pain and Juvenile Intra-Amygdalar CRF_1_ Blockade During Fear Conditioning

**FIGURE 5:**
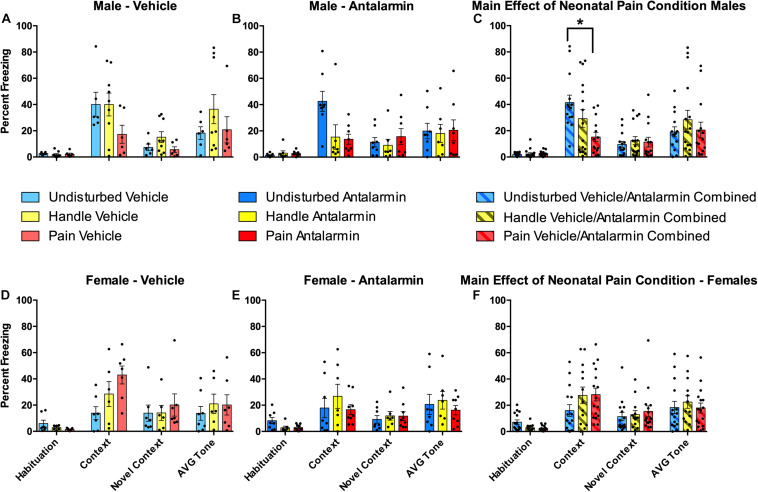
Effects of Antalarmin on freezing for neonatally manipulated rats during the four freezing variables (Habituation, Context, Novel Context, Average Tone) following fear conditioning in Experiment 1. The top three panels represent male Undisturbed, Handled, and Pain **(A)** vehicle (Undisturbed—N = 8; Handled—N = 12; Pain—N = 9) or **(B)** Antalarmin (Undisturbed—N = 11; Handled—N = 10; Pain—N = 10) treated rats. **(C)** The same data were also collapsed across drug to show the main effect of neonatal pain condition on freezing. The bottom three panels represent female Undisturbed, Handled, and Pain rats treated with **(D)** vehicle (Undisturbed—N = 7; Handled—N = 9; Pain—N = 8) or **(E)** Antalarmin (Undisturbed—N = 8; Handled—N = 9; Pain—N = 8), and **(F)** a panel with the same data collapsed across drug to show the main effect of neonatal pain condition on freezing. These results show that fear conditioning altered subsequent freezing for all groups. For females, vehicle-treated Pain rats had a statistically significantly higher freezing percentage during the Context test compared to Undisturbed vehicle-treated female rats (but not Handled) and no effect of Antalarmin treatment was found in females (all *p*’s > 0.10). In males, there was a statistically significant effect of neonatal pain condition only on the Context test which was reversed by Antalarmin. When data were collapsed across drug treatment, neonatal pain significantly lowered Context freezing compared to Undisturbed but not Handled males (all *p*’s > 0.1). Data are presented as means with error bars as ±SEM; *denotes significant (*p* < 0.05) difference between indicated groups.

Contextual freezing variables: Neonatal pain reduced freezing to the conditioning context in male rats (Figure 5C). This was shown by a 2 (Sex: Male, Female) × 2 (Drug: Vehicle, Antalarmin) × 3 (Condition: Undisturbed, Handle, Pain) repeated-measure ANOVA on the percent freezing during the first 5 min of the Habituation, Context, and Novel Context tests, which served as a repeated-measure “contextual freezing” variable, assessing baseline freezing, conditioned freezing expression to the conditioning apparatus, and generalization of freezing to a novel context, respectively. There was a statistically significant main effect of test session [*F* 2, 152) = 68.299, *p* < 0.001] and a statistically significant three-way interaction among the contextual freezing, condition, and sex variables [*F*(4, 152) = 6.32, *p* < 0.001]. The effect of test session merely confirms that fear conditioning alters subsequent freezing to the conditioning context, whereas the interaction is of interest to our hypothesis that neonatal pain effects subsequent behavior. To examine this interaction further, separate 2 (Drug: Vehicle, Antalarmin) × 3 (Condition: Undisturbed, Handle, Pain) repeated-measure ANOVAs were performed for each sex. In males, there was a statistically significant interaction between neonatal pain condition and freezing [*F*(3.03, 57.59) = 5.14, *p* = 0.001, Greenhouse–Geisser corrected]. Therefore, separate univariate ANOVAs were performed for each contextual freezing variable (Habituation, Context, and Novel Context). These analyses revealed a statistically significant effect of neonatal pain condition only on the Context test [*F*(2, 36) = 5.05, *p* = 0.01]. Post hocs revealed that Pain male rats (*p* = 0.006) but not Handled (*n.s.*) had reduced freezing on the Context test compared to Undisturbed rats (see Figure 5C). In females, there were no statistically significant main effects or interactions (all *p*’s > 0.1).

#### Effects of Neonatal Pain and Intra-Amygdalar CRF_1_ Blockade on Auditory Freezing

Neither neonatal pain nor CRF_1_ antagonism affected conditioned freezing to the auditory cue (Figure 5). A 2 (Sex: Male, Female) × 2 (Drug: Vehicle, Antalarmin) × 3 (Condition: Undisturbed, Handle, Pain) univariate ANOVA with the average percent freezing to the tone serving as the dependent variable resulted in non-statistically significant main effects or interactions (all *p*’s > 0.1).

#### Effects of Neonatal Pain and Intra-Amygdalar CRF_1_ Blockade on Subsequent Somatosensation

**FIGURE 6:**
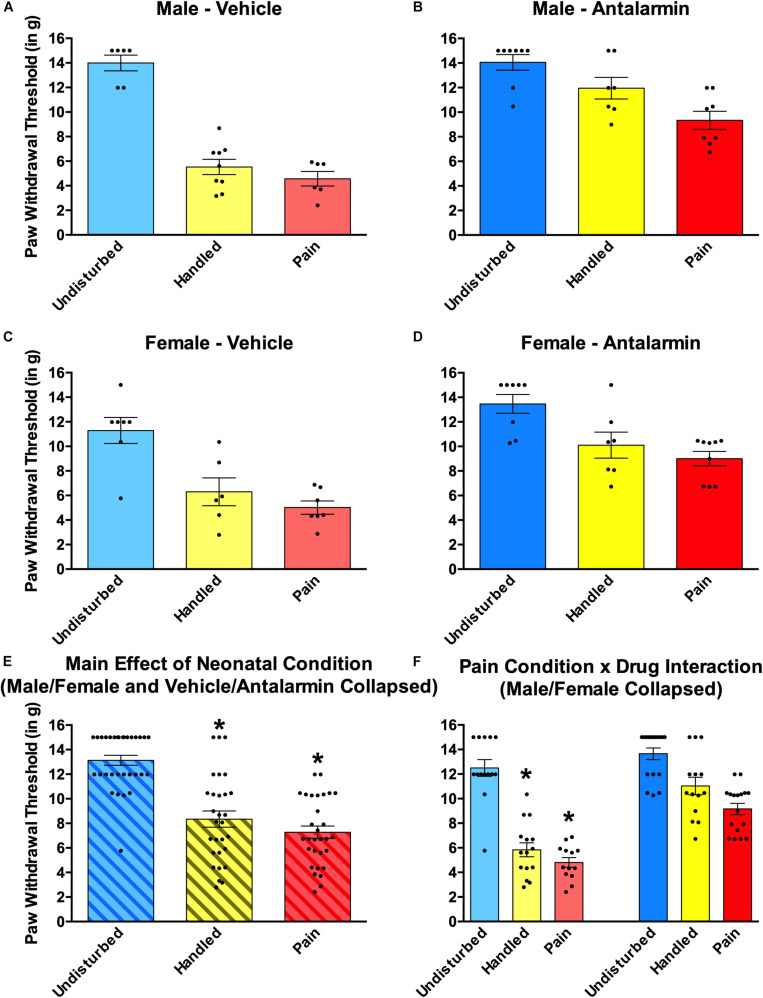
Effects of Antalarmin on paw withdrawal thresholds on the von Frey mechanical allodynia measure in neonatally manipulated rats for Experiment 1. Top two panels represent male, **(A)** vehicle-treated rats (Undisturbed—N = 8; Handled—N = 12; Pain—N = 9) and **(B)** Antalarmin-treated rats (Undisturbed—N = 11; Handled—N = 10; Pain—N = 10). The middle two panels represent female, **(C)** vehicle-treated rats (Undisturbed—N = 7; Handled—N = 9; Pain—N = 8), and **(D)** Antalarmin-treated rats (Undisturbed—N = 8; Handled—N = 9; Pain—N = 8). The bottom two panels represent **(E)** the same data collapsed across sex and drug treatment to show the main effect of neonatal condition and **(F)** the same data collapsed across sex but not drug to show the neonatal condition and drug interaction. These data indicated that neonatal manipulation creates a general effect of tactile hypersensitivity in both male and female rats, and this effect was reversed by Antalarmin treatment in both Handled and Pain conditions. Data are presented as means with error bars as ±SEM; *denotes significant (*p* < 0.05) difference between indicated groups.

Neonatal pain and handling produced robust tactile hypersensitivity that was reversed by intra-amygdalar administration of the CRF_1_ antagonist Antalarmin regardless of sex (Figure 6). This was shown by a 2 (Sex: Male, Female) × 2 (Drug: Vehicle, Antalarmin) × 3 (Condition: Undisturbed, Handle, Pain) univariate ANOVA with von Frey paw withdrawal threshold serving as the dependent measure. This resulted in a statistically significant main effect of condition [*F*(2, 76) = 67.97, *p* < 0.01] as well as a statistically significant interaction between neonatal pain condition and drug [*F*(2, 76) = 7.24, *p* < 0.01]. For the main effect, *post hocs* revealed that Pain (*p* < 0.001) and Handled (*p* < 0.001) rats had lower paw withdrawal thresholds than Undisturbed rats (see Figure 6E), but Handled and Pain rats did not differ significantly from one another (*n.s.*). To better understand the interaction, several separate univariate ANOVAs were run. First, separate 3 (Condition: Undisturbed, Handle, Pain) × 2 (Sex: Male, Female) ANOVAs were performed for each drug group, confirming that the neonatal manipulation produced an effect of condition in the Vehicle-treated [*F*(2, 35) = 55.40, *p* < 0.001], but not Antalarmin-treated subjects (*n.s*; see Figure 6F). This reversal was verified by conducting separate univariate 2 (Drug: Vehicle, Antalarmin) × 2 (Sex: Male, Female) ANOVAs for each neonatal pain condition (Undisturbed, Handle, Pain). These tests revealed that both Handled [*F*(1,25) = 31.79, *p* < 0.01] and Pain [*F*(1, 26) = 46.33, *p* < 0.01] but not Undisturbed (*n.*s) rats had a statistically significant effect of drug. Thus, Antalarmin treatment reversed the otherwise observed hypersensitivity (see Figure 6) in both neonatal pain conditions (Handled and Pain).

A 2 (Sex: Male, Female) × 2 (Drug: Vehicle, Antalarmin) × 3 (Condition: Undisturbed, Handle, Pain) univariate ANOVA with thermal paw withdraw latencies from the Hargreaves apparatus serving as the dependent variable revealed no statistically significant main effect of interactions (all *p*’s > 0.10; data not shown).

### Experiment 2—Effects of Neonatal Pain and Juvenile Intra-Amygdalar CRF_2_ Blockade During Fear Conditioning

**FIGURE 7:**
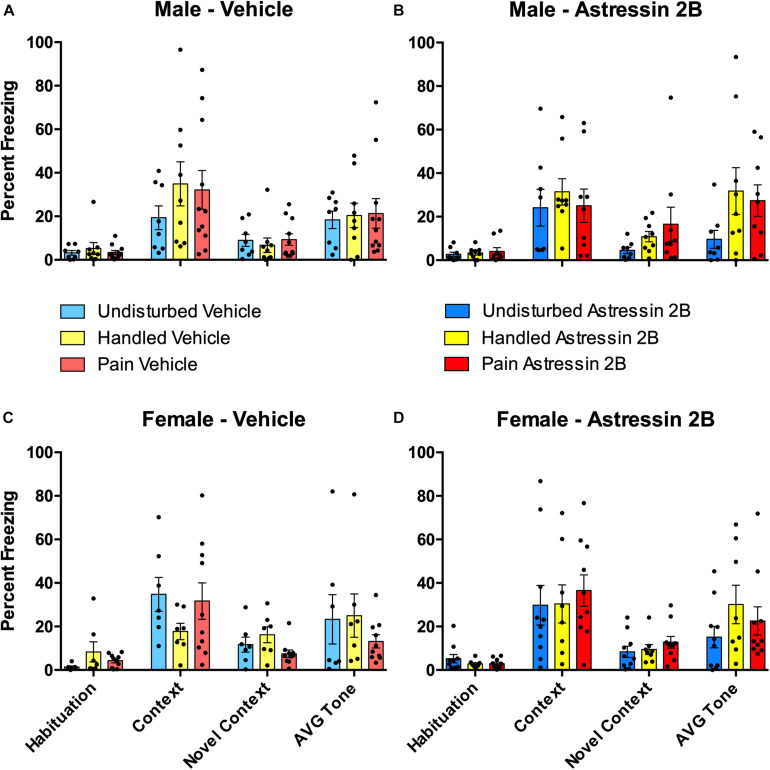
Effects of Astressin 2B on percent freezing for rats during the four freezing variables (Habituation, Context, Novel Context, Average Tone) following fear conditioning in neonatally manipulated rats for Experiment 2. Top two panels represent male Undisturbed, Handled, and Pain rats with **(A)** vehicle-treated rats (Undisturbed—N = 8, Handled—N = 9, Pain—N = 11) and **(B)** Astressin 2B-treated rats (Undisturbed—N = 8, Handled—N = 9, Pain—N = 9). The bottom two panels represent female Undisturbed, Handled, and Pain rats with **(C)** vehicle-treated rats (Undisturbed—N = 7, Handled—N = 7, Pain—N = 10) and **(D)** Astressin 2B-treated rats (Undisturbed—N = 10, Handled—N = 8, Pain—N = 10). These results show that fear conditioning altered subsequent freezing but show no other significance of condition or drug treatment. Data are presented as means with error bars as ±SEM.

The effects of neonatal pain and later-life CRF_2_ blockade on fear conditioning was analyzed via a 2 (Sex: Male, Female) × 2 (Drug: PND Vehicle, Astressin 2B) × 3 (Condition: Undisturbed, Handle, Pain) repeated-measure ANOVA on the percent freezing during the first 5 min of the Habituation, Context, and Novel Context tests, which served as a repeated-measure “freezing” variable (see Figure 7). As expected, there was a statistically significant main effect of test session [*F*(2, 121.41) = 90.77, *p* < 0.001, Greenhouse–Geisser corrected], again simply confirming that fear conditioning altered subsequent freezing. There were no other statistically significant main effects or interactions with these data (all *p*’s > 0.10).

#### Effects of Neonatal Pain and Intra-Amygdalar CRF_2_ Blockade on Auditory Freezing

Neither neonatal pain nor CRF_2_ antagonism affected conditioned freezing to the auditory cue (Figure 7). A 2 (Sex: Male, Female) × 2 (Drug: Vehicle, Astressin 2B) × 3 (Condition: Undisturbed, Handle, Pain) univariate ANOVA with the average percent freezing to the tone serving as the dependent variable resulted in no statistically significant main effects or interactions (all *p*’s > 0.1).

#### Effects of Neonatal Pain and Intra-Amygdalar CRF_2_ Blockade on Subsequent Somatosensation

**FIGURE 8:**
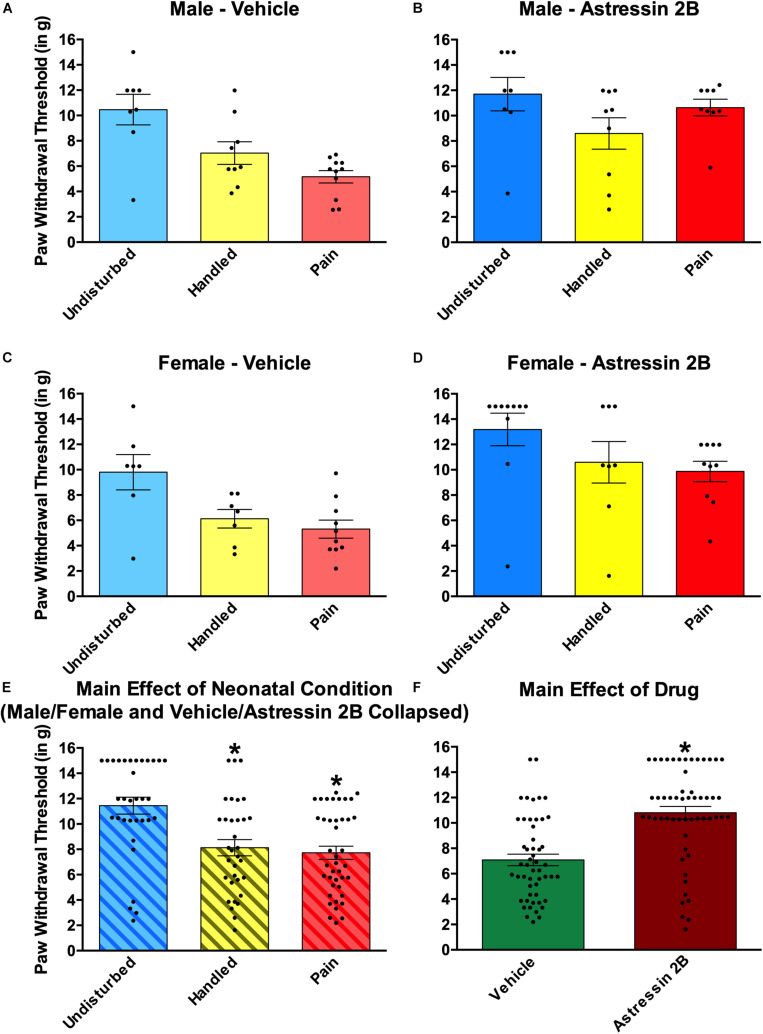
Effects of Astressin 2B on paw withdrawal thresholds on the von Frey mechanical allodynia measure in neonatally manipulated rats for Experiment 2. **(A)** Male vehicle-treated rats (Undisturbed—N = 8, Handled—N = 9, Pain—N = 11), **(B)** male Astressin 2B-treated rats (Undisturbed—N = 8, Handled—N = 9, Pain—N = 9), **(C)** female vehicle-treated rats (Undisturbed—N = 7, Handled—N = 7, Pain—N = 10), and **(D)** female Astressin 2B-treated rats (Undisturbed—N = 10, Handled—N = 8, Pain—N = 10). The bottom two panels depict **(E)** the same data collapsed across sex and drug to show the main effect of neonatal condition and **(F)** with the same data collapsed across sex and condition to show the main effect of drug. These data indicated that neonatal manipulation creates a tactile hypersensitivity in both male and female rats and that there is a general antinociceptive effect following CRF_2_ antagonism via Astressin 2B into the CeA. Data are presented as means with error bars as ±SEM; *denotes significant (*p* < 0.05) difference between indicated groups.

Neonatal pain and handling produced tactile hypersensitivity (see Figure 8). In addition, a general anti-nociceptive effect was observed following intra-amygdalar administration of the CRF_2_ antagonist Astressin 2B (see Figure 8). This was shown by a 2 (Sex: Male, Female) × 2 (Drug: Vehicle, Astressin 2B) × 3 (Condition: Undisturbed, Handle, Pain) univariate ANOVA with von Frey paw withdrawal threshold serving as the dependent measure. This resulted in a statistically significant main effect of Condition [*F*(2, 94) = 13.365, *p* < 0.001] and a statistically significant main effect of Drug [*F*(1,94) = 31.76, *p* < 0.001]. No statistically significant interactions were found. For the main effect of condition, *post hoc* analyses revealed that regardless of sex or drug, Handled and Pain rats displayed lower paw withdrawal thresholds compared to Undisturbed rats, but Handled and Pain rats did not differ significantly from one another (see Figure 8E). The main effect of drug revealed that Astressin 2B rats displayed higher paw withdrawal thresholds than Vehicle rats, regardless of sex or condition (see Figure 8F).

A 2 (Sex: Male, Female) × 2 (Drug: Vehicle, Astressin 2B) × 3 (Condition: Undisturbed, Handle, Pain) univariate ANOVA with thermal paw withdraw latencies from the Hargreaves apparatus serving as the dependent variable revealed no statistically significant main effect or interactions (all *p*’s > 0.05; data not shown).

### Experiment 3—Effects of Neonatal Pain on Juvenile Amygdalar CRF Expression

An outlier analysis was performed and consisted of excluding a subject’s single CRF measure if it was more than 2.5 standard deviations away from the mean for that condition on that measure. For CRF-positive cell count data in the CeA, there were seven outlying data points per hemisphere (9% of overall data points). For CeA CRF luminance data, there were three outlying data points per hemisphere (4% of overall data points). For CRF-positive cell count data in the BLA, there were six outliers per hemisphere (8% of overall data points). For BLA CRF luminance data, there was one outlier in the left (1% of left data points) and five outliers in the right (6% of right data points). Four to seven subjects remained in each condition. This high number of outliers (∼6% of total data points) is more than the ∼1.25% one would expect when using 2.5 SD as the standard and is likely attributed to variations in tissue quality, digestion, and background staining that occurred during processing. In particular, the protease step was one that required extensive optimization for our tissue.

#### Effects of Hemisphere, Sex, Juvenile Condition, and Neonatal Condition on c-Fos

Although staining was punctate and clear and our amygdala regions well defined, there were no apparent effects or trends of neonatal pain or fear conditioning on any c-fos measure in either region (BLA and CeA). This highly unexpected result suggests we missed the critical window for assessing c-fos expression. Therefore, we do not analyze or report these results further (see Supplementary Figure 1).

#### Effects of Hemisphere, Sex, Juvenile Condition, and Neonatal Condition on CeA CRF

**FIGURE 9:**
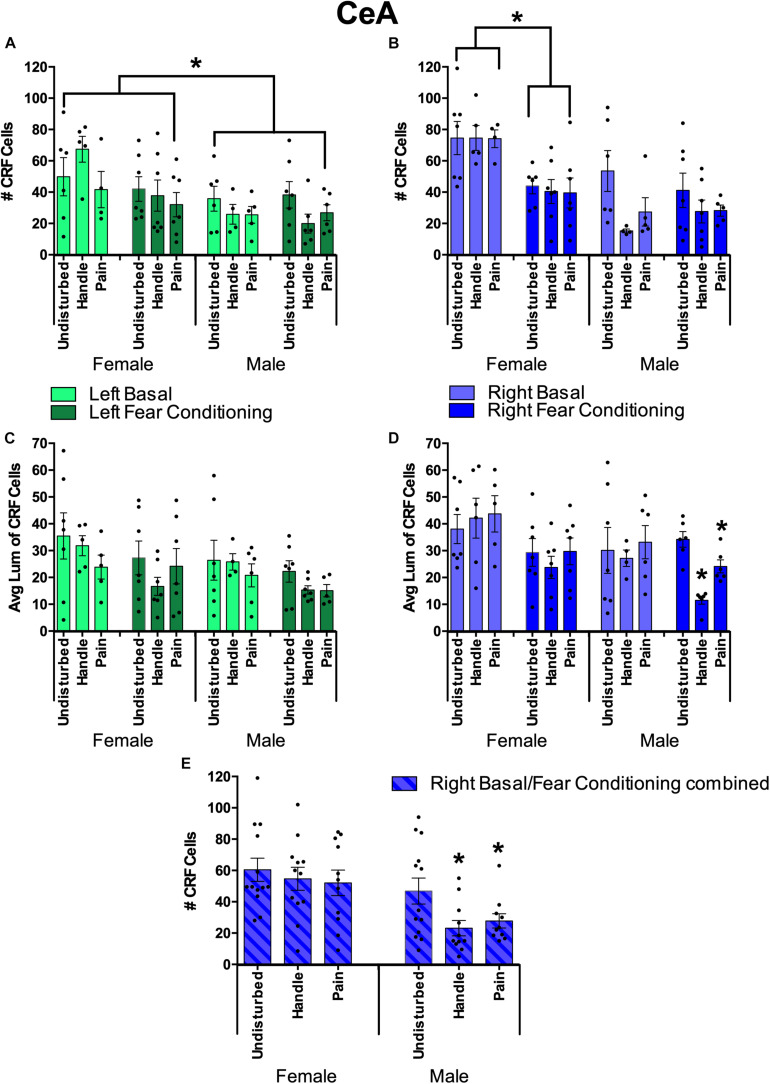
Quantification of FISH labeling for the Corticotropin Releasing Factor (CRF) in the Central Nucleus of the Amygdala (CeA) under neonatal conditions (Pain, Handled, and Undisturbed) and juvenile conditions (basal and fear-conditioned) in PND 24 rats. **(A)** Number of CRF + cells within the left CeA. **(B)** Number of CRF + cells within the right CeA. **(C)** Average luminance of CRF+ cells within the left CeA. **(D)** Average luminance of CRF+ cells within the right CeA. **(E)** Number of CRF + cells within the right CeA combined across juvenile condition. N’s ranged four to seven subjects per group in panels **(A–D)**. N’s ranged 11–13 subjects per group in panel **(E)**. Data are presented as means with error bars as ±SEM; *denotes significant (*p* < 0.05) difference between indicated groups.

In the CeA, neonatal pain or handling reduced CRF staining in a hemisphere-, sex-, and measure-dependent manner (Figure 9). RNAscope *in situ* hybridization analysis first consisted of 2(Sex: Male, Female) × 3(Neonatal condition: Undisturbed, Handled, Pain) × 2(Juvenile condition: Basal, Fear conditioned) × 2(Hemisphere: Right, Left) repeated-measure ANOVAs on two dependent variables (number of CRF+ cells and average luminance of CRF+ cells) to assess any effect of hemisphere for CRF measures in the CeA. There was a significant effect of hemisphere in the number of CRF-positive cells *F*(1,53) = 8.07, p = 0.006, and average luminance of CRF-positive cells *F*(1,59) = 22.40, p > 0.001, in the CeA. Therefore, subsequent univariate ANOVAs were performed separating hemispheres.

For the left hemisphere cell count, females had a greater number of CRF-expressing cells than males, although fear conditioning reduced the expression. This was shown by a significant main effect of sex in the number of CRF-positive cells [*F*(1, 58) = 10.74, *p* < 0.01] with females exhibiting more CRF-positive cells across groups (see Figure 9A). In addition, after separating sex, we found that females trended toward an effect of juvenile condition [*F*(1, 30) = 3.82, *p* = 0.06] with basal females exhibiting more CRF-positive cells than fear-conditioned ones. There were no observed significant effects or trends regarding CRF-positive cell count in males (*p*s > 0.10).

For left hemisphere CRF luminance, there was a significant main effect of juvenile condition [*F*(1, 62) = 4.97, *p* < 0.05] and a trend in sex [*F*(1,62) = 2.98, *p* = 0.089] in the average luminance of CRF-positive cells. Additionally, separating by sex showed that males trended toward an effect of juvenile condition [*F*(1,30) = 3.08, *p* = 0.09] with basal males possessing brighter left CeA CRF cells than fear conditioned ones.

For the right hemisphere cell count, neonatal stress reduced the number of CRF-expressing cells, but only in the CeA of male rats. This was shown by a significant main effect of sex [*F*(1,58) = 23.10, *p* < 0.001] and juvenile condition [*F*(1,58) = 9.47, *p* < 0.05] as well as a significant interaction between the two [*F*(1,58) = 9.80, *p* < 0.05]. Additionally, there was a trend toward a significant main effect of neonatal condition [*F*(2,58) = 2.68, *p* = 0.077] for the number of CRF-positive cells. Separating by sex found a significant effect of juvenile condition (fear-conditioned or not) in females [*F*(1,30) = 21.17, *p* < 0.001] with basal females displaying more CRF-positive cells than fear-conditioned ones (see Figure 9B). Additionally, there was a significant effect of neonatal condition in males [*F*(2,28) = 4.18, *p* < 0.05] with *post hoc* tests indicating that both male Handled (*p* = 0.015) and Pain (*p* = 0.05) subjects possessed fewer CRF-positive cells than Undisturbed males (Figure 9E).

For right-hemisphere luminance, there was a significant effect of sex [*F*(1,62) = 5.81, *p* < 0.05] and juvenile condition [*F*(1,62) = 10.22, *p* < 0.01]. When separated by sex, there was a significant effect of juvenile condition in females [*F*(1,33) = 8.85, *p* < 0.01] and a trend toward a significant effect of neonatal condition for males [*F*(2,29) = 2.81, *p* = 0.077]. When separated by juvenile condition, there was a significant effect in neonatal condition in juvenile fear-conditioned subjects [*F*(2,33) = 6.49, *p* < 0.01] with *post hoc* tests indicating lower CRF brightness in Handled (mean: 18.12; SEM: 2.86) vs Undisturbed (mean: 31.58; SEM: 3.05) animals (*p* < 0.01; see Figure 9). Additionally, there was a trend of sex [*F*(2,29) = 3.74, *p* = 0.063] observed in non-fear-conditioned (basal) subjects. Given these findings for CeA right-hemisphere luminance, an additional ANOVA was run separating both juvenile condition and sex. Male fear-conditioned subjects displayed a significant effect of neonatal condition [*F*(2,18) = 23.40, *p* < 0.001] with *post hoc* tests, indicating that Handled (*p* < 0.001) and Pain (*p* = 0.008) males had reduced brightness in right CeA CRF expression compared to Undisturbed males (Figure 9D), again consistent with reduced expression in neonatal pain and handled subjects.

#### Effects of Hemisphere, Sex, and Juvenile Condition on BLA CRF

**FIGURE 10:**
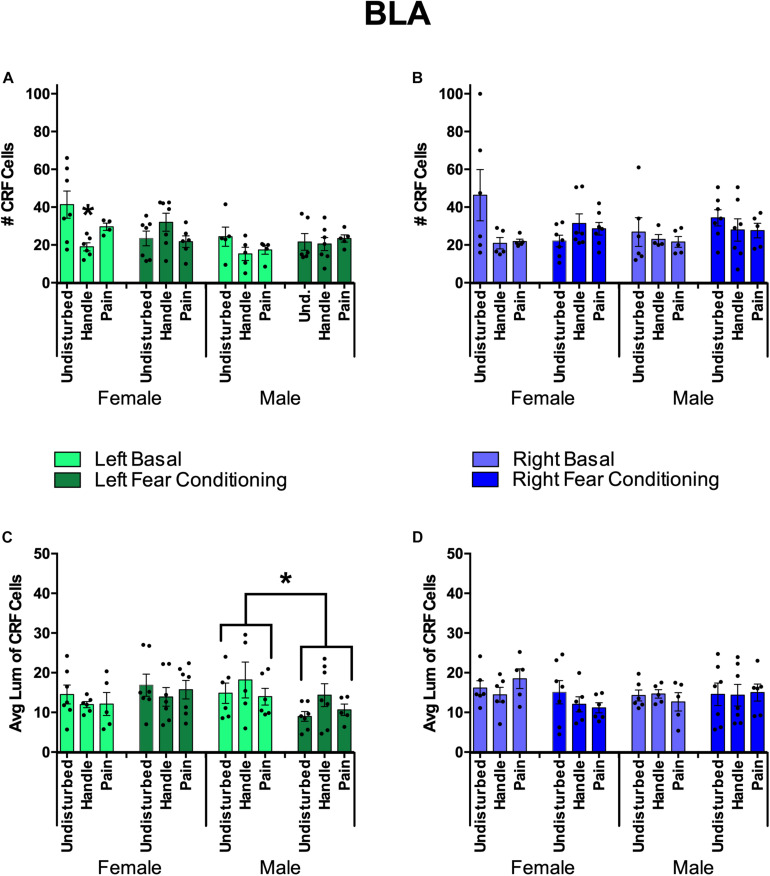
Basolateral Amygdala (BLA) Quantification of FISH Product Corticotropin Releasing Factor (CRF) under neonatal conditions (Pain, Handled, and Undisturbed) and juvenile conditions (basal and fear conditioned) in PND 24 rats. **(A)** Number of CRF+ cells within the left BLA. **(B)** Number of CRF+ cells within the right BLA. **(C)** Average luminance of CRF+ cells within the left BLA. **(D)** Average luminance of CRF + cells within the right BLA. N’s ranged four to seven subjects per group. Data are presented as means with error bars as ±SEM; *denotes significant (*p* < 0.05) difference between indicated groups.

In the BLA, neonatal pain or handling also reduced CRF staining in a hemisphere-, sex-, and measure-dependent manner (see Figure 10). Similar to the CeA CRF analysis, BLA CRF was first analyzed via 2(Sex: Male, Female) × 3(Neonatal condition: Undisturbed, Handled, Pain) × 2(Juvenile condition: Basal, Fear conditioned) × 2(Hemisphere: Right, Left) repeated-measure ANOVAs for two dependent variables (number of CRF+ cells and average luminance of CRF+ cells) to assess any effect of hemisphere for CRF measures in the BLA. There was a significant main effect of hemisphere in the number of CRF-positive cells [*F*(1,55) = 5.67, *p* < 0.05]. For the average luminance of BLA CRF, there was a significant interaction between hemisphere, sex, and juvenile condition [*F*(1,59) = 8.88, *p* < 0.01]. Given these findings, further univariate ANOVAs separating hemispheres were conducted.

For the right BLA, males and females displayed a trend toward a significant interaction between sex, neonatal condition, and juvenile condition [*F*(2,59) = 3.02, *p* = 0.056] in the number of CRF-positive cells (see Figure 10). No additional effects or trends were observed in right BLA CRF cell counts. In regard to average CRF luminance, there was a trend in the interaction of sex and juvenile condition [*F*(1,60) = 2.81, *p* = 0.099]. No other trends or effects were observed in right BLA CRF measures.

For the left BLA, fear conditioning reduced CRF expression in all animals. Additionally, females exposed to neonatal pain or handling displayed a further reduction in CRF expression. This was found by a significant effect of sex [*F*(1,58) = 8.67, *p* < 0.01] and a significant interaction of neonatal condition and juvenile condition [*F*(2,58) = 5.38, *p* < 0.01] in the number of CRF-positive cells. When separated by sex, there was a significant interaction between neonatal condition and juvenile condition in females [*F*(2,31) = 6.04, *p* < 0.01]. Given these findings, an additional univariate ANOVA was conducted separating subjects by sex and juvenile condition. There was a significant effect of neonatal condition for female basal subjects [*F*(2,14) = 4.80, *p* < 0.05]. *Post hoc* tests indicated that female basal Handled (*p* < 0.01) but not female basal Pain (*p* = 0.148) subjects had fewer CRF-positive cells compared to basal Undisturbed females (see Figure 10A). In regard to average CRF luminance, there was a significant interaction between sex and juvenile condition [*F*(1,63) = 5.85, *p* < 0.05]. When separated by sex, there was a significant effect of juvenile condition in male subjects [*F*(1,30) = 4.17, *p* = 0.05], indicating that basal male subjects displayed brighter CRF cells than fear-conditioned males (see Figure 10C). To conclude, when separated by juvenile condition, there was a significant effect of sex in fear-conditioned subjects [*F*(1,34) = 4.69, *p* < 0.05], indicating that fear-conditioned females possessed brighter CRF cells than fear-conditioned males (Figure 10).

## Discussion

The current experiments demonstrate sex-dependent changes in amygdalar CRF signaling in the lasting effects of neonatal trauma using a model that is representative of human NICU trauma. There are several notable aspects of the current results. First, these data confirm that neonatal pain alters subsequent tactile hypersensitivity. In addition, these data add to the literature by demonstrating that CRF signaling in the amygdala is enduringly altered by neonatal pain in lateralized and sex-dependent manner. Moreover, CeA CRF receptor signaling is required for the observed stress-induced hypersensitivity in neonatally manipulated rats. Specifically, Experiment 1 demonstrated that postweaning CRF_1_ antagonism during fear conditioning reverses the otherwise observed tactile hypersensitivity in rats that received neonatal pain or handling. In addition, Experiment 2 suggested that CRF_2_ antagonism produced a general antinociception, rather than a specific reversal of the fear conditioning-induced hypersensitivity. Finally, Experiment 3 showed that neonatal pain and handling produced a lateralized reduction in the number of cells expressing CRF. This occurred in the right CeA of male subjects and the left BLA of female subjects, consistent with previous evidence of sexual dimorphisms in the mechanisms of neonatal stress (Zuke et al., 2019).

As previously demonstrated, neonatal pain and handling produced a tactile hypersensitivity on PND 24 rats after fear conditioning (Davis et al., 2018), similar to the effects shown by others (Anand et al., 1999; Schwaller and Fitzgerald, 2014; Carmo et al., 2016; Chen et al., 2016; Davis and Burman, 2020). The reversal by CRF_1_ antagonism demonstrated that amygdalar CRF signaling was critical for this effect, consistent with its known role in pain in adults (Rouwette et al., 2012) including arthritis (Ji and Neugebauer, 2007; Neugebauer et al., 2020) and neuropathic pain (Andreoli et al., 2017). This is also consistent with prior data showing that Antalarmin reversed a hypersensitivity induced by a gastric suctioning procedure on neonatal rats (Smith et al., 2007) as well as the effects of CRF_1_ antagonism on the lasting effects of pre-weaning odor-shock pairing (Prusator and Greenwood-Van Meerveld, 2017). It should be noted that Antalarmin was administered systemically at the time of neonatal trauma whereas in the current study the drug was administered into the CeA prior to the activating trauma on PND 24.

CRF receptor 2 antagonism using Astressin 2B produced a general antinociception across all of the neonatal treatment groups. This effect was particularly surprising considering previous research with CeA CRF_2_ antagonism, which suggests that CRF_2_ blockade failed to play a major role in amygdalar pain modulation (Fu and Neugebauer, 2008). Additionally, research has shown that CRF_2_ *activation* is necessary for the analgesic on/off switching of amygdalar function (Rouwette et al., 2012), and likewise, CRF-induced analgesia was prevented by the systemic co-administration of Astressin 2B (Yarushkina et al., 2009), which appears to be the opposite of our findings. Ji and Neugebauer (2008) demonstrated that CRF administration facilitates cellular responding to a noxious stimulus in the amygdala, and a CRF_1_ antagonist (but not a CRF_2_ antagonist) reversed this effect, whereas the cellular inhibition induced by a higher dose of CRF was blocked by CRF_2_ antagonism. In contrast to the previous literature, but consistent with our findings, Kokkotou et al. (2006) showed that CRF_2_ knockout mice have reduced inflammatory pain responses. Thus, while the literature is divergent, there is some prior evidence that disruption of CRF_2_ can produce antinociception.

Our lab previously demonstrated that neonatal (PD 6) males, but not females, had increased CRF expression within the amygdala immediately following neonatal pain (Zuke et al., 2019). We now show that neonatal stress causes a long-term decrease of amygdalar CRF expression into adolescence. This suggests that developmental changes following neonatal pain-induced CRF expression may lead to a downregulation in the number of CRF-positive cells within the CeA or BLA later in life. This is consistent with previous work on neonatally stressed degus (Becker et al., 2007). Moreover, there is some evidence in adult rats that CRF plays a non-monotonic role in pain, with slight increases in concentration producing a pro-nociceptive effect while large increases in concentration produce an anti-nociceptive effect (Ji and Neugebauer, 2008). Thus, a reduction in the number of CRF cells in the amygdala may cause a leftward shift in CRF expression and be a mechanism for the stress-induced hypersensitivity that we observe.

However, these effects may be specific to the timing and modality of the neonatal stress, as well as the sex of the subject. Previous studies have linked gonadal hormone modulation in adult rats that experienced unpredictable early life trauma (odor/shock pairings during the 2nd week of life) with subsequent visceral hypersensitivity in adult intact females (but not males). Gonadectomies in females resulted in a male-like phenotype (i.e., abolished visceral hypersensitivity), while estradiol replacement reestablished hypersensitivity (Chaloner and Greenwood-Van Meerveld, 2013b). This study implicates gonadal hormones and sex differences in the development of pain following early life adversity (for additional review, see Chaloner and Greenwood-Van Meerveld, 2013a). To add to this, this sex difference appears to be modulated by epigenetic modulation of amygdalar CRF and its interactions with GR expression (Prusator and Greenwood-Van Meerveld, 2017; Louwies and Greenwood-Van Meerveld, 2020).

The current study observed a strong effect of lateralization for all CRF measures within the CeA, with the right hemisphere exemplifying the greatest differences between groups in male subjects and some indication that the left hemisphere was altered in females. This is consistent with other research indicating that the right CeA primary responds to nociception (Neugebauer et al., 2004; Allen et al., 2021). In male subjects, our data demonstrated that neonatal stress reduced the number of CRF-positive cells only within the right CeA. Further, neonatally undisturbed males had brighter CRF cells in the right CeA compared to Pain and Handled males exposed to our secondary fear-conditioning stressor. Female subjects showed a different pattern, consistent with the known sexual dimorphism of neonatal stress. Within the right CeA, female rodents subjected to fear conditioning had fewer CRF-expressing cells compared to basal females. This may be the result of fear conditioning causing an increase in CeA CRF translation resulting in diminished CRF mRNA levels. Indeed, there is evidence of increased activation of the CeA (Baker and Kim, 2004; Li et al., 2013) and rapid CRF translation during stress (Jurek et al., 2015), especially when the stressor is novel (Aguilera and Liu, 2012).

Less robust to the effects observed in the CeA, we also observed lateralization of the effects of early life pain on CRF in the BLA of females. Interestingly, and unlike the CeA, these effects were predominately limited to the left hemisphere which is primarily recognized for processing positive emotions and stimuli (Neugebauer et al., 2004; Ji and Neugebauer, 2009; Allen et al., 2021). Within the left BLA, basal females showed a reduction in the number of CRF-expressing cells following neonatal handling compared to Undisturbed controls. Thus, the reduction in the number of CRF cells following neonatal stress appears to be consistent but occurs in different regions depending on sex.

Thus, only in the right CeA of males did we find that neonatal stress leads to a reduction in CRF expression in the juvenile period. In the right CeA of females, we found that fear conditioning during the juvenile period reduced CRF expression regardless of neonatal condition. Finally, in the left CeA, we observed a reduction of CRF expression in females exposed to non-painful neonatal stress. We view these patterns as largely consistent with previous findings that CRF-containing cells in the right CeA are responsible for processing painful and stressful stimuli (Neugebauer et al., 2004; Allen et al., 2021) and add to this literature by suggesting that neonatal pain alters their function. Previous research indicates the left hemisphere and left CeA as processors of positive emotions and stimuli (Neugebauer et al., 2004; Ji and Neugebauer, 2009; Allen et al., 2021); however, our data expands upon this by suggesting that the left amygdala may have a role in processing early life stressors in females.

We have previously reported that neonatal pain alone can disrupt auditory fear conditioning later in life (Davis et al., 2018, 2020; Davis and Burman, 2020). However, in this experiment, there were no reliable effects of the neonatal pain on later freezing across experiments. This is likely due to the use of a weaker conditioned stimulus (0.3 mA), which lowered freezing levels close to the behavioral floor, in order to allow the assessment of potential bidirectional changes caused by the CRF antagonists as well as the implementation of a surgery on PND 21, which may have masked any potential effect.

Future work should address several aspects of this study. First, that both CRF_1_ and CRF_2_ antagonisms were anti-nociceptive was surprising. These findings merit replication using a variety of doses and more specific drugs to ensure both the replicability and specificity of the effects. Although our antagonist doses are commonly used in the literature (Henry et al., 2006; Gondré-Lewis et al., 2016; Zorrilla et al., 2002), it is also possible that our observations represent a general disruption of amygdalar function (due to CRF receptor antagonism), which may not occur at lower doses. Second, the lack of c-Fos activation by fear conditioning was surprising, although the literature on amygdalar c-fos expression is mixed (see Campeau et al., 1991; Rosen et al., 1998). We believe that we may have missed the critical peak of c-fos expression after fear conditioning. However, in other stress studies measuring CRF and c-fos mRNA expression, an increase in c-fos expression did not correlate in any effect with CRF (Kovács, 2008), suggesting that this is unlikely to influence any of our CRF findings. Additional work using a different time course or a different immediate early gene (perhaps EGR-1; see Deal et al., 2016) may be useful.

Taken together, the current experiments confirm that neonatal stress influences pain sensitivity later in life and begins the process of determining the neural substrates underlying this effect. Neonatal pain causes sex-dependent changes in the amygdala CRF system that persist into the postweaning period, and this effect appears to be lateralized. The vulnerability to tactile hypersensitivity produced by neonatal pain exposure requires CRF_1_ activation to manifest. Amygdalar CRF_2_ antagonism prior to an activating stressor appears to produce a general antinociception, an effect which requires additional study. Nevertheless, the amygdalar CRF system appears to be a promising target for future studies on neonatal stress and trauma.

## Data Availability Statement

The raw data supporting the conclusions of this article will be made available by the authors, without undue reservation.

## Ethics Statement

The animal study was reviewed and approved by University of New England Institutional Animal Care and Use Committee.

## Author Contributions

SD, JZ, and MBe were the primary authors involved in data collection, analysis, and composition of the manuscript. MBu is the PI and provided intellectual oversight, feedback and editing necessary for completing the manuscript. All authors contributed to the article and approved the submitted version.

## Conflict of Interest

The authors declare that the research was conducted in the absence of any commercial or financial relationships that could be construed as a potential conflict of interest.

## Supplementary Material

Supplementary Figure 1 Quantification of FISH Product c-fos within the amygdala under neonatal conditions (Pain, Handled, and Undisturbed) and juvenile conditions (basal and fear conditioned) in PND 24 rats. **(A)** Number of c-fos positive cells within CeA. **(B)** Number of c-fos positive cells in the BLA. N’s ranged 4–7 subjects per group. Data are presented as means with error bars as ±SEM.
