# Supplementary figures and images for: Amygdalar Corticotropin-Releasing Factor Signaling Is Required for Later-Life Behavioral Dysfunction Following Neonatal Pain
Source: Front Physiol. 2021 May 11;12:660792. doi: 10.3389/fphys.2021.660792 (PMC8144524; doi:10.3389/fphys.2021.660792)

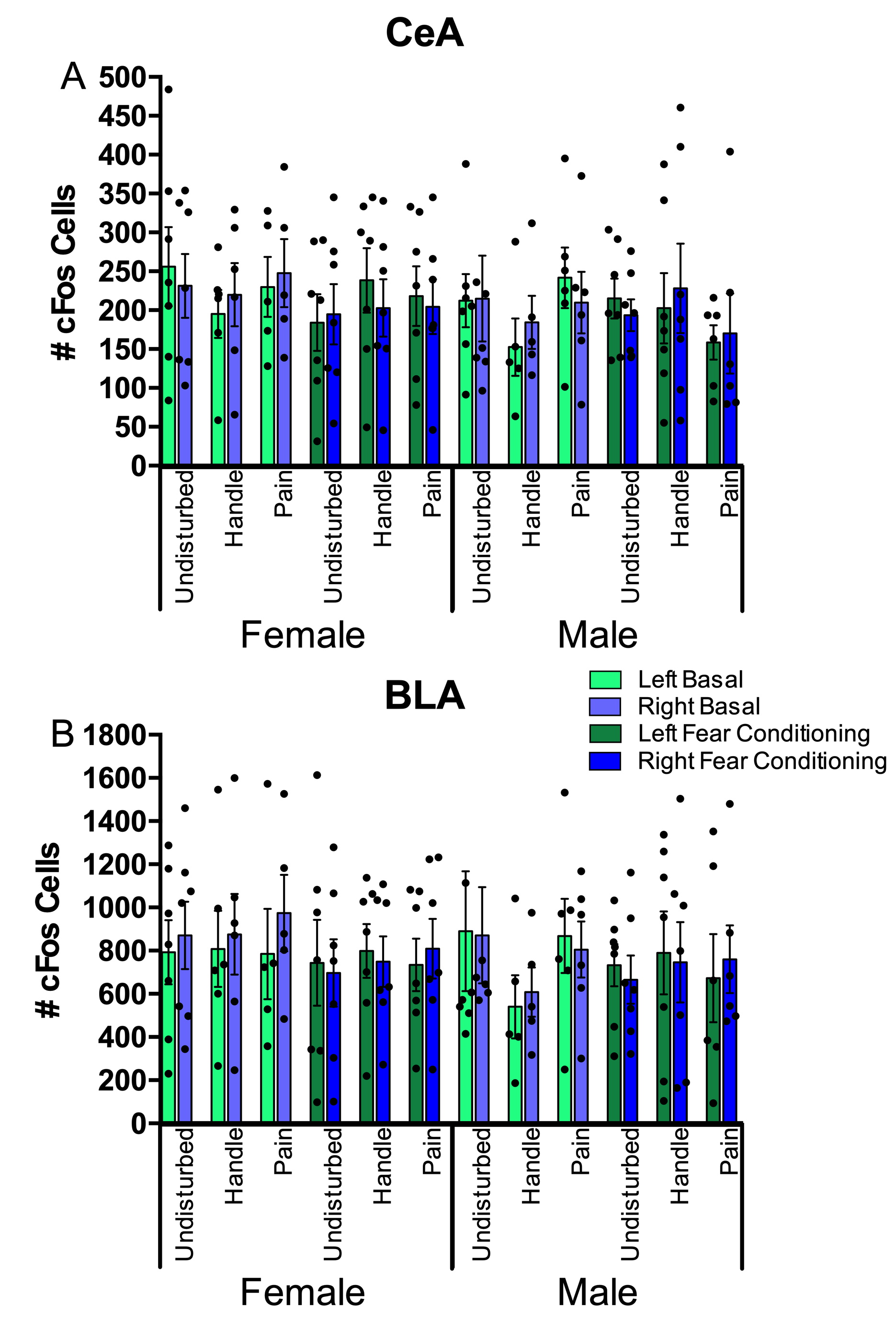

Supplement: Supplementary Figure 1 — Quantification of FISH Product c-fos within the amygdala under neonatal conditions (Pain, Handled, and Undisturbed) and juvenile conditions (basal and fear conditioned) in PND 24 rats. (A) Number of c-fos positive cells within CeA. (B) Number of c-fos positive cells in the BLA. N’s ranged 4–7 subjects per group. Data are presented as means with error bars as ±SEM. [file Image_1.TIFF]
